# Interaction of Intercellular Adhesion Molecule 1 (*ICAM1*) Polymorphisms and Environmental Tobacco Smoke on Childhood Asthma

**DOI:** 10.3390/ijerph110606504

**Published:** 2014-06-20

**Authors:** Yu-Fen Li, Che-Chen Lin, Chien-Kuo Tai

**Affiliations:** 1 Institute of Biostatistics, China Medical University, 91 Hsieh-Shih Rd., Taichung 404, Taiwan; E-Mail: yufenli@mail.cmu.edu.tw; 2 Department of Public Health, China Medical University, 91 Hsieh-Shih Rd., Taichung 404, Taiwan; E-Mail: u9665023@cmu.edu.tw; 3 Department of Life Science, National Chung Cheng University, 168 University Rd., Min-Hsiung Township, Chia-Yi County 621, Taiwan

**Keywords:** ICAM1, environmental smoke exposure, childhood asthma

## Abstract

Asthma is a chronic disease that is particularly common in children. The association between polymorphisms of the gene encoding intercellular adhesion molecule 1 (ICAM1) and gene-environment interactions with childhood asthma has not been fully investigated. A cross-sectional study was undertaken to investigate these associations among children in Taiwan. The effects of two functional single-nucleotide polymorphisms (SNPs) of *ICAM1*, rs5491 (K56M) and rs5498 (K469E), and exposure to environmental tobacco smoke (ETS) were studied. Two hundred and eighteen asthmatic and 877 nonasthmatic children were recruited from elementary schools. It was found that the genetic effect of each SNP was modified by the other SNP and by exposure to ETS. The risk of asthma was higher for children carrying the rs5491 AT or TT genotypes and the rs5498 GG genotype (odds ratio = 1.68, 95% confidence interval 1.09–2.59) than for those with the rs5491 AA and rs5498 AA or AG genotypes (the reference group). The risk for the other two combinations of genotypes did not differ significantly from that of the reference group (*p* of interaction = 0.0063). The two studied *ICAM1* SNPs were associated with childhood asthma among children exposed to ETS, but not among those without ETS exposure (*p* of interaction = 0.05 and 0.01 for rs5491 and rs5498, respectively). Both *ICAM1* and ETS, and interactions between these two factors are likely to be involved in the development of asthma in childhood.

## 1. Introduction

Asthma is a common chronic inflammatory disease worldwide. The prevalence of asthma increased between 2001 and 2010, and worldwide premature deaths from this disease have been estimated at 250,000 per year [1]. The prevalence of asthma has been increasing worldwide [2,3], including Taiwan [4]. A study conducted in Ireland suggested that the symptoms of severe asthma had increased among children aged 13–14 years between 1995 and 2007 [5]. A consequence of the increasing prevalence of asthma has been an associated increase in costs; for example, the cost associated with asthma in the USA increased from about U.S. $53 billion in 2002 to about U.S. $56 billion in 2007 [6]. Moreover, it has been reported that asthma prevalence was higher among children than adults [1].

Asthma often causes airflow obstruction, bronchial hyper responsiveness, and chronic inflammation, and is a complex disorder that is caused by several factors, including genetic and environmental. The environmental factors associated with the development of asthma are quite complex. For example, it is well known that the nicotine and other chemicals present in environmental tobacco smoke (ETS) can be delivered to the fetus via the placenta if a mother smokes during pregnancy [7,8], and any resulting effects can be transgenerational [9]. Some air pollutants are also associated with the development of asthma, including ozone [10,11], NO_2_ [12], and fine particulate matters [13,14]. The interactions between the genetic and environmental factors associated with asthma have also been studied previously [11,15,16,17].

The gene encoding human intercellular adhesion molecule 1 (ICAM1) is associated with an inflammatory response, with the encoded protein being expressed on the cell membrane of several cell types including endothelial cells, epithelial cells, and fibroblasts. ICAM1 is the most important receptor of human rhinovirus infection [18], and some studies have found that asthma can also be induced by rhinovirus infection [19,20,21]. Many single-nucleotide polymorphisms (SNPs) of *ICAM1* have been reported, and some of these SNPs, such as rs5491 (K56M) and rs5498 (K469E), are now known to be associated with asthma [22,23,24]. However, the association between *ICAM1* SNPs/haplotypes and childhood asthma has not yet been studied previously in a Chinese population.

The aims of this study were to determine: (1) the association between *ICAM1* SNPs and childhood asthma, and (2) the effect of any interaction between exposure to ETS and *ICAM1* SNPs on asthma risk. 

## 2. Materials and Methods

### 2.1. Study Design

School-aged children in Taiwan were recruited between April 2006 and April 2008 to investigate their respiratory illnesses and associated factors. The participants were elementary-school students in northern, central, and southern regions of Taiwan. A structured questionnaire, which was modified from the Chinese translated version of the International Study of Asthma and Allergies in Childhood core questionnaire, was completed by the participants’ parents or guardians with their informed consent. Buccal cell samples were then obtained from each of the children as a source of their DNA. This study was approved by the Ethics Review Committee of China Medical University.

### 2.2. Ascertainment of the Presence of Asthma and Exposure to ETS

Asthma was defined according to the parents’ or guardians’ answer to the question “Has a doctor ever diagnosed your child as having asthma?” The current number of household smokers (categorized into 0, 1, 2, and 3 or more), and whether the mothers had smoked during pregnancy were also recorded.

### 2.3. DNA Collection and Genotyping

Buccal scrapes were collected using a standard protocol, and genomic DNA was isolated using Gentra Puregene DNA isolation kits (Gentra Systems, Minneapolis, MN, USA), similar to the procedure described for the Children’s Health Study [25]. Genotyping of *ICAM1* polymorphisms was performed using commercially available premade TaqMan genotyping assays (Applied Biosystems, Foster City, CA, USA). Each sample was subjected to 50 amplification cycles on an ABI GeneAmp PCR System 9700 (Applied Biosystems). The resulting fluorescent signals of the two probes were analyzed using an ABI PRISM 7900HT Sequence Detection System (Applied Biosystems).

### 2.4. Statistical Analyses

The distributions of categorical variables were compared between asthmatic and nonasthmatic children using chi-square tests. Agreement with the Hardy-Weinberg equilibrium was assessed using a chi-square goodness-of-fit test. The genetic model of the *ICAM1* rs5498 polymorphism (A→G) was selected based on Akaike’s information criterion, and the two less-common rs5491 genotypes (AT and TT) were combined for the analysis due to the frequency of the minor allele (T) being small. The *ICAM1* haplotypes were estimated using the TagSNPs program provided by Dr. Stram [26].

Associations were reported in terms of odds ratios (ORs) and 95% confidence intervals (CIs), which were estimated with logistic regression models adjusted for gender, grade, parental education level, and the school location. Hosmer and Lemeshow test was used to assess the goodness-of-fit of models. The cutoff for statistical significance was set at *p* < 0.05. All analyses were conducted using SAS software (version 9.3, Cary, NC, USA).

## 3. Results

Selected characteristics of the participants are stratified according to asthma status in Table 1. In total, 218 asthmatic and 877 nonasthmatic children with complete genotype information for both of the studied *ICAM1* SNPs were included in the statistical analysis. The elementary-school boys were more likely to have asthma than their female counterparts (62.84% *vs.* 48.69%, *p* = 0.0002). While the asthma prevalence did not differ with grade (*p* = 0.1367), children with a higher parental education level had a higher risk for asthma (*p* = 0.0001). Maternal smoking during pregnancy was rare in both the asthmatic and nonasthmatic groups (0.46% *vs.* 1.48%). Although there were more current household smokers in the nonasthmatic group, those smokers appeared to avoid smoking inside the house when they had an asthmatic child. Breastfeeding history was not associated with asthma (*p* = 0.4225). Children with asthma were more likely to suffer from allergic rhinitis and eczema (*p* < 0.0001), in line with the accepted knowledge that childhood asthma is linked with the family history of allergic diseases. Nevertheless, the distributions of both of the studied *ICAM1* SNPs did not differ significantly between the asthma and nonasthmatic groups (*p* = 0.5724 and 0.6876 for rs5491 and rs5498, respectively).

**Table 1:** Selected characteristics of participants by asthma status.

Variables		Asthmatics (n = 218)	Nonasthmatics (n = 877)	*p*-value
		n (%)	n (%)	
**Demographic characteristics**				
Gender				
	Boys	137 (62.84)	427 (48.69)	0.0002
	Girls	81 (37.16)	450 (51.31)	
Grade				
	1–2	85 (39.00)	281 (32.05)	0.1367
	3–4	88 (40.36)	382 (43.55)	
	5–6	45 (20.64)	214 (24.40)	
Parental education level				
	Graduate school and above	38 (17.43)	99 (11.29)	0.0001
	University or college	126 (57.80)	420 (47.89)	
	Senior high school or less	52 (23.85)	353 (40.25)	
	Missing values	2 (0.92)	5 (0.57)	
**Exposure Status**				
Maternal smoking during pregnancy				
	No	213 (97.71)	858 (97.83)	0.1379
	Yes	1 (0.46)	13 (1.48)	
	Missing values	4 (1.83)	6 (0.68)	
Numbers of household smokers				
	None	131 (60.09)	426 (48.57)	0.0058
	One	60 (27.52)	332 (37.86)	
	Two and above	25 (11.47)	117 (13.34)	
	Missing values	2 (0.92)	2 (0.23)	
Breastfed				
	No	89 (40.83)	400 (45.61)	0.4225
	Yes	126 (57.80)	468 (53.36)	
	Missing values	3 (1.38)	9 (1.03)	
**Allergic diseases**				
Lifetime allergic rhinitis				
	No	43 (19.72)	457 (52.11)	<0.0001
	Yes	171 (78.44)	402 (45.84)	
	Missing values	4 (1.83)	18 (2.05)	
Lifetime eczema				
	No	122 (55.96)	658 (75.03)	<0.0001
	Yes	86 (39.45)	184 (20.98)	
	Missing values	10 (4.59)	35 (3.99)	
**Family history of allergic diseases**				
Family history of eczema				
	No	132 (60.55)	614 (70.01)	0.0012
	Yes	77 (35.32)	206 (23.49)	
	Missing values	9 (4.13)	57 (6.50)	
Family history of asthma				
	No	158 (72.48)	757 (86.32)	<0.0001
	Yes	52 (23.85)	77 (8.78)	
	Missing values	8 (3.67)	43 (8.78)	
Family history of allergic rhinitis				
	No	78 (35.78)	402 (45.84)	0.0021
	Yes	133 (61.01)	422 (48.12)	
	Missing values	7 (3.21)	53 (6.04)	
*ICAM1* polymorphisms *				
rs5491 (A→T) genotype				
	AA	194 (88.99)	794 (90.54)	0.5724
	AT	24 (11.01)	81 (9.24)	
	TT	0	2 (0.23)	
rs5498 (A→G) genotype				
	AA	122 (55.96)	466 (53.14)	0.6876
	AG	83 (38.07)	348 (39.68)	
	GG	13 (5.96)	63 (7.18)	

Note: * Genotypes in controls were in Hardy-Weinberg equilibrium (*p* = 0.965 and 0.858 for rs5491 and rs5498, respectively).

The genotype and haplotype associations between *ICAM1* polymorphism and asthma—after adjusting for gender, grade, parental education level, school location, and exposure to ETS—are presented in Table 2. As demonstrated by the unadjusted analysis given in Table 1, neither of the studied *ICAM1* SNPs exerted a main effect on asthma. The risk for asthma was slightly higher among children carrying the rs5491 AT or TT genotypes (OR = 1.12, 95% CI = 0.89–1.41) than those with the rs5498 GG genotype (OR = 1.08, 95% CI = 0.77–1.52). However, there was a significant interaction effect between rs5491 and rs5498 on asthma (*p* = 0.0063). Compared with children who simultaneously carried the rs5491 AA and the rs5498 AA or AG genotypes, the risk for asthma was significantly higher among those with the rs5491 AT or TT genotype and the rs5498 GG genotype (OR = 1.68, 95% CI = 1.09–2.59). However, the risk was lower for children carrying the other two combinations of genotype. The rs5491 and rs5498 *ICAM1* haplotypes were also studied. Two of the four haplotypes (T-A and T-G in the order of rs5491–rs5498) were rare, and no significant haplotype effects were observed.

**Table 2:** The association between *ICAM1* polymorphism and asthma.

**rs5491**	**n**	**aOR**	**95% CI**
AA	988	ref	ref
AT + TT	107	1.12	0.89–1.41
**rs5498**	**n**	**aOR**	**95% CI**
AA+AG	1019	ref	ref
GG	76	1.08	0.77–1.52
rs5491	n	aOR	95% CI
AA	938	ref	ref
AA	50	0.61	0.35–1.07
AT + TT	81	0.97	0.74–1.27
AT + TT	26	1.68	1.09–2.59
*p* for the interaction = 0.0063			
Haplotype *	**Frequency **	**aOR**	**95% CI**
A-G	0.218	ref	ref
A-A	0.732	0.96	0.84–1.11
T-A	0.002	1.38	0.56–3.44
T-G	0.048	1.03	0.82–1.30

Notes: aOR: adjusted odds ratio; CI: confidence interval; Models were adjusted for gender, grade, parental education level, school location, and the exposure of environmental tobacco smoke; * Haplotypes were in the order of rs5491–rs5498. ref: reference.

**Figure 1:**
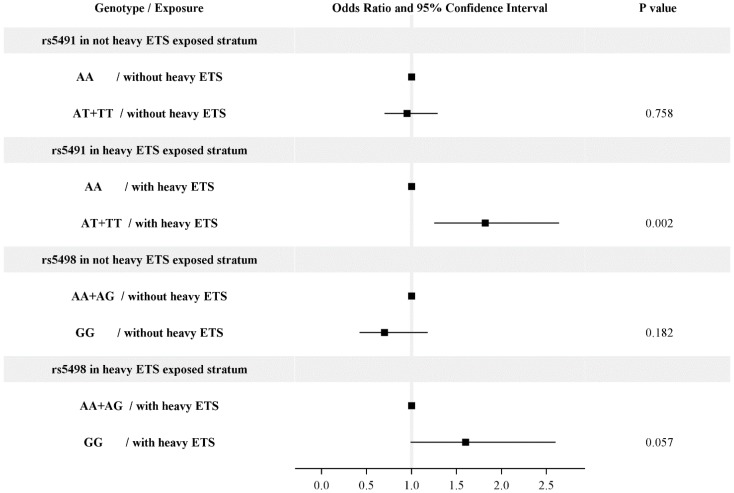
The interaction of exposure to environmental tobacco smoke (ETS) with rs5491 or rs5498 for asthma development. Models were adjusted for gender, grade, parental education level, and school location. Exposure to heavy ETS was defined as having more than one household smokers. The effect of rs5491 and rs5498 was modified by ETS (*p* = 0.05 and 0.01, respectively).

The risk for asthma associated with risk genotype was much higher in children exposed to heavy ETS (Figure 1). Exposure to heavy ETS was defined as having more than one household smoker. Among children exposed to heavy ETS, the risk for asthma was more than 1.8 higher among those carrying the rs5491 AT or TT genotype than those carrying the rs5491 AA genotype (OR = 1.82, 95% CI = 1.25–2.64). Similarly, the risk for asthma was higher among children carrying the rs5498 GG genotype than for those carrying the rs5498 AA or AG genotype (OR = 1.60, 95% CI = 0.99–2.6). However, the risk genotypes were not significantly associated with asthma among children not exposed to heavy ETS. The effects of rs5491 and rs5498 were thus modified by heavy ETS exposure (*p* = 0.05 and 0.01, respectively). Hosmer and Lemeshow goodness-of-fit tests demonstrated the models considering the SNPs interaction and haplotype were well fitted (*p* = 0.4308 and 0.8212, respectively). 

## 4. Discussion and Conclusions

In the present study it was found that the two investigated *ICAM1* SNPs (rs5491 and rs5498) did not exert a main effect on childhood asthma. However, the genetic effect of each SNP was modified by both the other SNP and exposure to ETS.

The boys in the present study had a higher risk for asthma, which is consistent with the results of other epidemiological studies of asthma in Taiwan [27,28]. The finding of children with a higher parental education level at a higher risk for asthma was also observed in other studies [11,29]. One possible explanation is that parents with higher education level are more aware of asthma as suggested in a recent report [30]. The allele frequencies of rs5491 and rs5498 in our data were similar to the Chinese data in the HapMap project and the results of another two Chinese studies [31,32]. Both of the studied *ICAM1* SNPs were functional SNPs, so they could influence the encoded enzyme [23,24,33].

**Figure 2:**
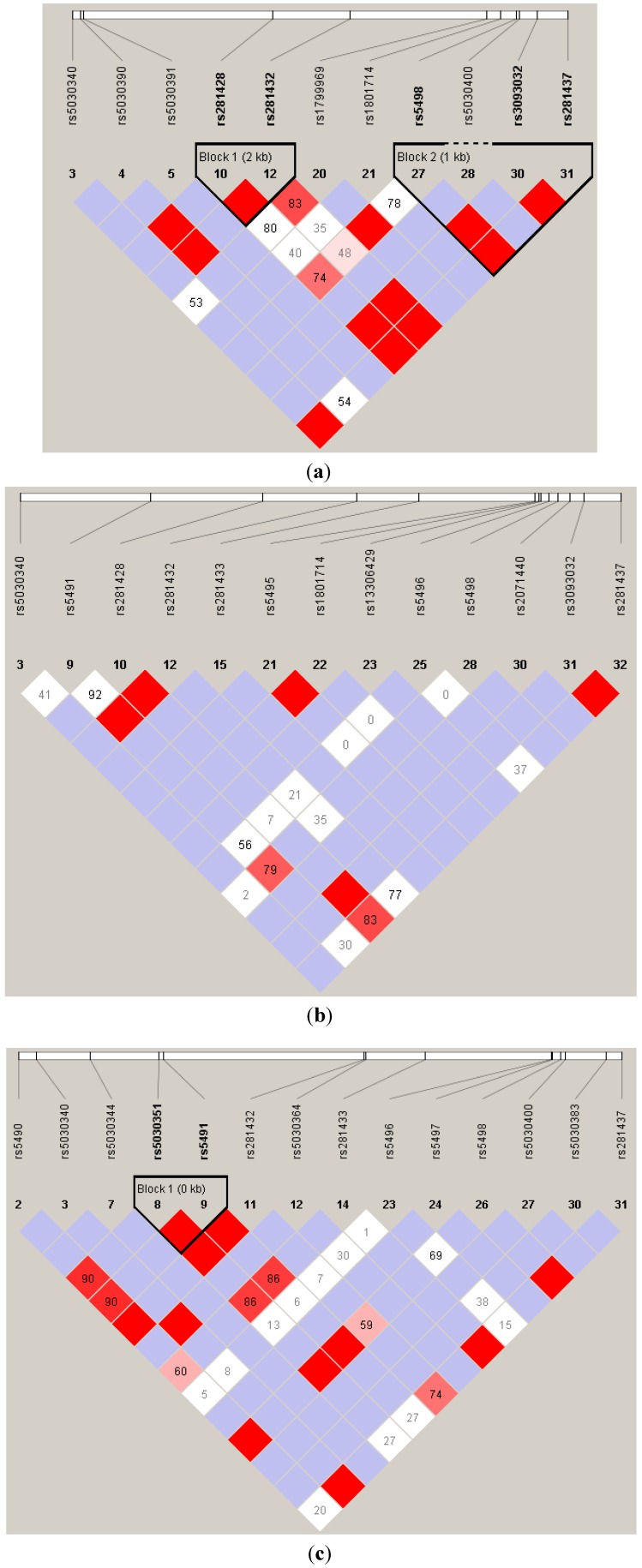
The likage equalibrium of *ICAM1* single-neuclitide polymorphsms (SNPs) by different race populations: (**a**) CEU (Utah residents with Northern and Western European ancestry), (**b**) CHB (Han Chinese in Beijing, China), and (**c**) YRI (Yoruban in Ibadan, Nigeria). The minor allele frequency (MAF) of the two studied SNPs (rs5491 and rw5498) are shown. Data was dowloaded from HapMap (Data Rel 27 PhaseII + III on NCBI B36 assembly, dbSNP b126).

Notes: (**a**) MAF(rs5491) = 0 and MAF(rs5498) = 0.384; (**b**) MAF(rs5491)=0.078 and MAF(rs5498)=0.244; (**c**) MAF(rs5491)=0.250 and MAF(rs5498)=0.182.

It has been shown that ICAM1-deficient mice exhibit less inflammatory-cell infiltration in the lungs and a reduction in airway hyperresponsiveness [34]. Furthermore, Stanciu and Djukanovic demonstrated that when inflammatory responses occurred, white blood cells migrated to the sites of inflammatory responses through epithelial cells [35], and then white blood cells interacted with adhesion molecules on the epithelial cells. The ligand for ICAM1 was lymphocyte function-associated antigen 1 (LFA-1) [35,36]. The structural change in the N-terminal domain of ICAM1 caused by the rs5491 T allele, which decreases the binding capacity of the protein to the LFA-1, fibrinogen, and human rhinovirus, may be responsible for the reduced the risk for asthma among carriers [36]. However, Li *et al.* indicated that the risk for asthma was higher in rs5491 T allele carriers [22]. The ICAM1 rs5498 A allele had been reported to be positively associated with childhood asthma in African-Americans [22], Germans [23], and the Dutch [24]. The *ICAM1* haplotype of rs5493 (G241S) and rs5498 [22], and another haplotype of rs5498 and rs885743 (in the 3’-untranslated region) [23] were reported to be associated with the risk for childhood asthma. Nevertheless, our data revealed no significant association between both genotypes and haplotypes of rs5491 and rs5498, and asthma. To the best of our knowledge, no association studies have shown an interaction between *ICAM1* and ETS in the context of asthma development. However, Grigg *et al.* found that the soluble ICAM1 level of bronchoalveolar lavage was markedly higher in children whose parents smoked than in those whose parents did not [37]. Sarecka-Hujar *et al.* reported a synergistic interaction between the rs5498 genotype and smoking that affected the risk of coronary artery disease [38]. All of these results support the present findings. These results discrepancies between studies might be due to racial differences in the study populations or unobserved confounding and effect modifications. As shown in Figure 2, the linkage equilibrium of ICAM1 SNPs are different by racial population. Especially, rs5491 is not polymorphic in CEU. Moreover, there was a significant gene–gene interaction between rs5491 and rs5498, and a gene-environment interaction between ETS and both rs5491 and rs5498 in the present study but not mentioned in other studies. 

Since this study had a cross-sectional design, it was subject to some limitations. The temporal or causal relationships between environmental factors and asthma development could not be established. Moreover, recall bias may have been present due to the use of a self-reported questionnaire. Lack of any control for heterogeneous diagnosis of asthma and age of diagnosis of asthma was also a limitation of the study because no detailed information was available. Furthermore, population stratification [39] might be a confounder in genetic association studies. Almost all of the students in the present study were Han Chinese. Han Chinese in Taiwan can be divided into three major subgroups: Minnan, Hakka, and Mainlanders, although Yang *et al.* found no differences in the genomic structure between these three subgroups [40]. Thus, we consider that the population stratification had little influence in this study. Last but not least, we cannot rule out the possibility of false positives under the issue of multiple testing.

*ICAM1* plays a role in asthma development. Among those children who were exposed to ETS, the risk of asthma development was higher for those carrying the rs5491 AT or TT genotypes and for those carrying the rs5498 GG genotype. Functional analyses are required to further clarify the pathophysiological mechanism conferred by *ICAM1* polymorphisms and ETS exposure.
